# Genome‐wide identification of chitin‐binding proteins and characterization of BmCBP1 in the silkworm, *Bombyx mori*

**DOI:** 10.1111/1744-7917.12552

**Published:** 2018-02-04

**Authors:** Zhi‐Lang Li, Sha Tian, Huan Yang, Xia Zhou, Shu‐Ping Xu, Zi‐Yu Zhang, Jing Gong, Yong Hou, Qing‐You Xia

**Affiliations:** ^1^ State Key Laboratory of Silkworm Genome Biology College of Biotechnology Southwest University Chongqing China

**Keywords:** *Bombyx mori*, chitin, cuticle protein, metamorphosis, molting

## Abstract

The insect cuticle plays important roles in numerous physiological functions to protect the body from invasion of pathogens, physical injury and dehydration. In this report, we conducted a comprehensive genome‐wide search for genes encoding proteins with peritrophin A‐type (ChtBD2) chitin‐binding domain (CBD) in the silkworm, *Bombyx mori*. One of these genes, which encodes the cuticle protein BmCBP1, was additionally cloned, and its expression and location during the process of development and molting in *B. mori* were investigated. In total, 46 protein‐coding genes were identified in the silkworm genome, including those encoding 15 cuticle proteins analogous to peritrophins with one CBD (CPAP1s), nine cuticle proteins analogous to peritrophins with three CBD (CPAP3s), 15 peritrophic membrane proteins (PMPs), four chitinases, and three chitin deacetylases, which contained at least one ChtBD2 domain. Microarray analysis indicated that CPAP‐encoding genes were widely expressed in various tissues, whereas PMP genes were highly expressed in the midgut. Quantitative polymerase chain reaction and western blotting showed that the cuticle protein BmCBP1 was highly expressed in the epidermis and head, particularly during molting and metamorphosis. An immunofluorescence study revealed that chitin co‐localized with BmCBP1 at the epidermal surface during molting. Additionally, *BmCBP1* was notably up‐regulated by 20‐hydroxyecdysone treatment. These results provide a genome‐level view of the chitin‐binding protein in silkworm and suggest that BmCBP1 participates in the formation of the new cuticle during molting.

## Introduction

The cuticle forms the insect exoskeleton, which covers the surface of the insect body and plays important roles in growth control, environmental protection and wound healing. During development, the insect cuticle undergoes several rounds of molting to overcome size limitations (Petkau *et al*., 2012). The cuticle is mainly formed from chitin, small amounts of lipids and cuticle proteins, which blend with chitin to form the natural architecture of biomacromolecules that protect the insect's body (Togawa *et al*., 2004; Deng *et al*., 2016). To date, several chitin‐binding cuticle proteins have been identified and characterized in various insect species (Andersen *et al*., 1995; Hamodrakas *et al*., 2002; Togawa *et al*., 2004; Iconomidou *et al*., 2005; Futahashi *et al*., 2008; Petkau *et al*., 2012; Mun *et al*., 2015; Deng *et al*., 2016).

A comparison of the cuticle proteins from various species indicates that some of these proteins possess consensus chitin‐binding domains (Rebers & Riddiford, 1988; Rebers & Willis, 2001). To date, two major families of chitin‐binding domains (CBDs), the ChtBD2 domain and the R&R domain (Rebers & Willis, 2001; Jasrapuria *et al*., 2012; Tetreau *et al*., 2015), have been identified in insect cuticle proteins via sequencing studies. The ChtBD2 domain belongs to the CBM14 family of carbohydrate‐binding domains (Chang & Stergiopoulos, 2015). The consensus sequence motif, which comprises six cysteine residues of the ChtBD2 domain in insects, has been defined as CX15‐17CX5‐6CX9CX12CX 6–7C (Tellam *et al*., 1999). This was recently modified to CX11‐30CX5‐6CX9‐24CX12‐17CX6‐12C following a genome‐wide search of chitin‐binding domains from *Manduca sexta* (Tetreau *et al*., 2015).

ChtBD2 was discovered in the insect peritrophic membrane proteins (PMPs); therefore, proteins with the ChtBD2 motif used to be referred to as “peritrophins” and the ChtBD2 motif was termed the peritrophin A‐type domain (Tellam *et al*., 1999; Jasrapuria *et al*., 2010). An analysis of the sequence features of PMPs shows that the CBDs of these proteins have conserved cysteine residues or non‐polar amino acid residues, which might have similar functionality for interacting with chitin/proteins (Wang & Granados, 2001). To date, most proteins with the ChtBD2 motif have been identified in the peritrophic membrane of insect species; examples include peritrophin‐44 and peritrophin‐48 of *Lucilia cuprina* (Elvin *et al*., 1996; Schorderet *et al*., 1998), Ag‐Aper1 of *Anopheles gambiae* (Shen & Jacobs‐Lorena, 1998), and peritrophin‐57 and peritrophin‐37 of *Spodoptera litura* (Chen *et al*., 2014). These peritrophins within the ChtBD2 domain and the conserved cysteine in the ChtBD2 domain are linked by disulfide bridges to form a binding pocket, in which conserved hydrophobic residues form hydrogen bonds with chitin fibrils. The ChtBD2 domain may influence PM structure and properties, as some peritrophins may interact only with specific chitin conformations (Hegedus *et al*., 2009). The multiple ChtBD2s of PMPs enable the assembly of proteins and chitin fibrils to form membranous structures supported by chitin fibrils in the PM (Wang & Granados, 2001).

Besides the peritrophins, ChtBD2 was also discovered in the obstructor family of invertebrates, which contained three ChtBD2 domain and a signaling peptide (Behr & Hoch, 2005). Obstructor‐A in *Drosophila* has been considered to play an important role for packaging of protein and chitin matrix in apical cells and is necessary for extracellular matrix in cuticle forming organs (Petkau *et al*., 2012; Pesch *et al*., 2015). Obstructor‐E has been reported as an important protein that controls the oriented contractility/expandability in *Drosophila* (Tajiri *et al*., 2017). The completion of genome sequencing projects for numerous species has facilitated the analysis of species at the genome level. Jasrapuria *et al*. (2010) conducted a genome‐wide bioinformatics search of the genes encoding ChtBD2‐containing proteins in the genome of *Tribolium castaneum*, and classified these genes into three main families. In addition, Tetreau *et al*. (2015) performed an exhaustive search for genes encoding ChtBD2‐containing proteins in the genome of *M. sexta*, and found 53 genes encoding 56 chitin‐binding proteins, containing CPAP1s, CPAP3s, PMPs, chitinase, chitin deacetylases. Members of the CPAP family exhibited differential spatial expression patterns and are widely expressed in various cuticle‐forming tissues.

In this study, we performed a genome‐level search to identify genes encoding chitin‐binding proteins containing ChtBD2 in the silkworm, and examined the expression pattern of these proteins in silkworm tissues. Furthermore, BmCBP1, one of the ChtBD2‐containing proteins of the silkworm, was characterized and its expression and localization studied during silkworm development and ecdysis.

## Materials and methods

### Insects and reagents

The silkworm strain *Dazao* was used in the present study. The silkworm was reared at a temperature of 25 ± 1°C on mulberry leaves. The 4th and 5th instar larvae were used for dissection to isolate the cuticle and various tissues for analyses. RNA extraction was performed using the Total RNA kit (Omega Bio‐Tek, Norcross, GA, USA) according to the manufacturer's instructions. The p28 vector (a pET28a‐derived vector), T4 DNA ligase, and restriction enzymes were purchased from TaKaRa (Otsu, Japan). All polymerase chain reaction (PCR) primers (Table 1) were synthesized by Shanghai Sangong Co. Ltd. (Shanghai, China).

**Table 1:** Polymerase chain reaction (PCR) primers used in this study

Name	Nucleotide sequence (5′ to 3′)
Complementary DNA clone	
BmCBP1‐p28‐*Nde* I F	5′ GGGAATTCCATATGCAAGAATTCAAATGCCCGGAC 3′
BmCBP1‐p28‐*Xho* I R	5′ CCGCTCGAGTTACGCCTTCTTGGGAAGCTTGT 3′
Quantitative PCR analysis	
QBmCBP1 F	5′ AGTACTAGGGTTGGCTGTTTGTGG 3′
QBmCBP1 R	5′ CAGTGTTCTTTGTTGGGGTTTTCA 3′

### Identification of genes encoding chitin‐binding proteins in the B. mori genome

The identification of genes encoding chitin‐binding proteins was performed according to a previously reported method (Tetreau *et al*., 2015). We conducted an extensive search of the silkworm genome (SilkDB, http://www.silkdb.org/silkdb/) and National Center for Biotechnology Information (NCBI) (http://www.ncbi.nlm.nih.gov/protein/) to identify all proteins predicted to contain the peritrophin A‐type (ChtBD2‐type) domain (pfam01607). Protein sequences with the ChtBD2 chitin‐binding domain (CBD) from *Drosophila* and other insects were downloaded from NCBI (http://www.ncbi.nlm.nih.gov/protein/) and utilized as queries to identify genes encoding proteins with ChtBD2s in *B. mori*. The protein sequences for *B. mori* identified in the initial search were used as queries for a second round of Basic Local Alignment Search Tool (BLAST) search to identify additional proteins with ChtBD2. This process was repeated until no additional proteins with the ChtBD2 domain could be identified.

Based on their sequence homology and expression distribution in different tissues, the identified proteins with CBD domain identified are classified into five different classes, including CPAP1s, CPAP3s, PMPs, chitinase, and chitin deacetylases.

### Whole‐genome microarray analysis of expression of silkworm ChtBD2 genes

Oligonucleotide microarray data were acquired from the SilkDB (http://www.silkdb.org/microarray/search.php) for a total of 35 genes from ten different tissues (Xia *et al*., 2007). Gene expression in multiple silkworm tissues from the 3rd day of the 5th instar larvae of *Dazao* was then investigated. Hierarchical clustering of gene expression patterns was performed using HemI software, as previously reported (Deng *et al*., 2014).

### Protein sequence and phylogenetic analyses

Protein sequence analysis was used to predict molecular weight (MW) and isoelectric point (pI) using the ExPASy proteomics website (http://web.expasy.org). Conserved domains in the protein sequence were identified via NCBI (http://www.ncbi.nlm.nih.gov/) and SilkDB (http://www.silkdb.org/silkdb/). Multiple sequence alignments of proteins were performed using ClustalX (http://clustal-x.software.informer.com/1.8/) and GENEDOC software (http://genedoc.software.informer.com/). ClustalX software was also used to perform multiple sequence alignments prior to phylogenetic analysis. MEGA6.0 software (http://www.megasoftware.net/mega6) was used to construct the phylogenetic tree using the neighbor‐joining method. To assess the branch strength of the phylogenetic tree, a bootstrap analysis of 2000 replications was performed. Bootstrap values of no less than 20% are shown on each branch of all trees generated. The identification of genes encoding chitin‐binding proteins was performed according to a previously reported method (Tetreau *et al*., 2015).

### Bioinformatics Analysis of the BmCBP1 promoter

Bioinformatics analysis of *cis*‐regulatory elements was performed on the upstream 2.0‐kb promoter sequences of *BmCBP1* genes, which were obtained from the silkworm genome database (http://www.silkdb.org/silkdb/). Potential ecdysone response elements (EcREs) were predicted via the JASPAR CORE database (http://jaspar.genereg.net/) using the elements of *Drosophila*, such as Eip74EF and EcR::usp.

### Production and purification of recombinant BmCBP1 and preparation of polyclonal antibody

To obtain the complementary DNA (cDNA) sequence of *BmCBP1*, total RNA was extracted from the epidermis of 3rd day 5th instar larvae using the Total RNA kit (Omega Bio‐Tek, Norcross, GA, USA). Two primers (Table 1) were then used to amplify the open reading frames (ORFs). The PCR products obtained as a result of the amplification of the ORFs were inserted into the protein expression vector p28, between the *Eco*R I and *Bam*H I restriction sites. *Escherichia coli* BL21 (DE3) cells were transformed with the recombinant plasmid, and protein expression was induced by adding 1.0 mmol/L isopropyl β‐D‐1thiogalactopryranoside at 37°C. The His‐tagged fusion protein was purified using Ni‐resins (Novagen, Madison, WI, USA). The Bradford assay was used for protein quantification (Bradford, 1976). The fusion BmCBP1 proteins were used for production of polyclonal antibodies by ZeHeng Biotech (Chongqing, China).

### Real‐time quantitative PCR and western blot analysis

Total RNA was extracted from various tissues at the prepupal stage, as well as from the epidermal tissues from the 4th instar larval stage to the 1st day of moth, using the Total RNA kit (Omega Bio‐Tek, Norcross, GA, USA). Extracted total RNA was then treated with RNase‐free Dnase I (TaKaRa, Otsu, Japan). Then, 3 μg of messenger RNA (mRNA) was transcribed into single‐strand cDNAs by first‐strand cDNA synthesis. Reverse transcription was performed using the reverse transcription Moloney Murine Leukemia Virus kit (Omega Bio‐Tek, Norcross, GA, USA) according to the manufacturer's protocol. Real‐time quantification PCR was performed to check the tissue specificity and expression pattern of various developmental stages, using pairs of gene‐specific primers of which are listed in Table 1. Real‐time quantification PCR was performed using a Premix Ex Taq TMII SYBR RT‐PCR kit (TaKaRa, Otsu, Japan) and an ABI Prism 7500 Step One PlusTM Real‐Time PCR System (Applied Biosystem, California, USA). The mRNA relative expression level of *BmCBP1* was analyzed using the 2^−ΔΔCt^ methods. Triplicate experiments were conducted for each sample. The statistical analyses were performed using non‐paired *t*‐test. The epidermis was isolated from silkworm tissues from the 4th instar larval stage to the 1st day of moth. The samples were homogenized in protein extraction buffer (8 mol/L urea, 1 mmol/L dithioerythritol and 10 mmol/L Chaps) and vortexed at 4°C for 2 h. Samples were then centrifuged (Eppendorf) at 10 000 × *g* at 4°C for 10 min. Supernatants containing soluble proteins were stored at −80°C. The Bradford assay was used for protein quantification.

Protein samples (20 μg) were mixed with 10 × loading buffer (250 mmol/L Tris‐HCl [pH 6.8], 10% SDS, 0.5% (Bromophenol blue) [BPB], 50% glycerol and 5% β‐mercaptoethanol) and then resolved at 120 V for approximately 80 min on 12% SDS‐PAGE (polyacrylamide gel electrophoresis). After SDS‐PAGE, the proteins were transferred onto a poly(vinylidene difluoride) membrane. The membrane was blocked in 5% non‐fat milk in TBS (20 mmol/L Tris base [pH 7.5], 140 mmol/L NaCl) for 1 h, Then, the membrane was incubated with anti‐BmCBP1 antibody (1 : 20 000) and a secondary goat anti‐rabbit antibody (1 : 40 000), diluted 1 : 1 000 in 1% non‐fat milk for 1 h at 37°C, and washed three times with TBST buffer (TBS + 0.05% Tween‐20) for 10 min each time. Secondary antibodies were detected using Renaissance Western blot Chemiluminescence Reagent Plus (New England Nuclear, Boston, MA, USA).

### Ecdysteroid treatment

To survey whether 20‐hydroxyecdysone (20E) affects the expression of the *BmCBP1* gene, 20E was dissolved (10 mg/mL) and diluted to working concentrations with dimethyl sulfoxide (DMSO). Solutions containing 1 μg, 2.5 μg, 5 μg or 10 μg of 20E (Sigma, St Louis, MO, USA) were injected into the silkworm via the spiracle of the 2nd day of 5th instar larvae. The same volume of DMSO was injected into the larvae as a control. After 24 h, the epidermal tissue from each treatment was dissected on ice, and immediately stored in liquid nitrogen for real‐time quantitative PCR analysis. The statistical analyses were conducted using non‐paired *t*‐test.

### Immunofluorescence

Immunofluorescence co‐localization of chitin and BmCBP1 was performed as described by Deng *et al*. (2016). Sections of cuticles (thickness, 5 μm) were prepared. The sections were blocked for 1 h in 20 mmol/L phosphate‐buffered saline (PBS) containing 5% bovine serum albumin. The sections were then incubated with anti‐BmCBP1 antibodies at a dilution of 1 : 300 for 1 h at 37°C, then washed three times with PBST (PBS + 0.5% Triton‐X100) buffer for 10 min each. Then, the sections were incubated with Cy3‐conjugated anti‐rabbit immunoglobulin G (1 : 1000 in blocking buffer) as the secondary antibody for 1 h at room temperature, followed by washing with PBST three times. Next, fluorescein isothiocyanate‐conjugated wheat germ agglutinin (WGA 1 : 100; Sigma, St Louis, MO, USA) chitin‐binding probe was applied and incubated at room temperature for 1 h. The sections were washed with PBS three times, for 10 min each, and then the nuclei were stained with 4',6‐diamidino‐2‐phenylindole dihydrochloride (DAPI) at a dilution of 1 : 1000, for 30 min. After washing three times in PBS buffer, the sections were observed and photographed under a fluorescence microscope (Olympus FV500, Tokyo, Japan).

## Results

### Identification and characterization of proteins with the ChtBD2 domain

When ChtBD2‐containing proteins from *Drosophila melanogaster* and other insects were queried against the silkworm genome via a BLAST search, a total of 46 proteins containing at least one ChtBD2 domain were identified in *B. mori*. The proteins with identified ChtBD2 domains are classified into five different classes based on their sequence homology and tissue specificity of gene expression from the *B. mori* microarray database. These included 15 CPAP1s, nine CPAP3s, 15 PMPs, four chitinases, and three chitin deacetylases (Table 2). Compared with the CPAP family, the PMP family of proteins exhibits a much larger variation in terms of the number of ChtBD2s, which ranges from one to 42 CBDs, while chitinases and chitin deacetylases contain both the ChtBD2 domain and a catalytic domain.

**Table 2:** Genes encoding proteins with ChtBD2 domains in *Bombyx mori*

	Gene name	SilkDB accession #	Protein length (aa)	Molecular weight (kDa)	Isoelectric point	Chitin‐binding domain	Scaffold	Starting point	End point	Chain
**Cuticular proteins analogous to peritrophins (CPAPS)**	*CPAP1‐A*	BGIBMGA009891‐PA	1902	211.9	5.1	1	nscaf2970	1189518	1203068	+
	*CPAP1‐B*	BGIBMGA009892‐PA	600	67.3	7.5	1	nscaf2970	1254824	1258803	+
	*CPAP1‐C*	BGIBMGA003270‐PA	267	30.1	4.6	1	nscaf2623	1089104	1093561	+
	*CPAP1‐D*	BGIBMGA006382‐PA	680	77.4	6.1	1	nscaf2853	4057195	4062514	−
	*CPAP1‐E*	BGIBMGA003272‐PA	136	15.5	4.8	1	nscaf2623	1140325	1145284	+
	*CPAP1‐F*	BGIBMGA010029‐PA	1254	140.1	5.2	1	nscaf2983	584122	590871	−
	*CPAP1‐G*	BGIBMGA003273‐PA	1004	117.8	8.7	1	nscaf2623	1199941	1214980	+
	*CPAP1‐H*	BGIBMGA001052‐PA	183	20.9	5.7	1	nscaf1898	1844898	1855609	−
	*CPAP1‐I*	BGIBMGA000421‐PA	702	78.3	9.3	1	nscaf1681	2799619	2802417	+
	*CPAP1‐J*	BGIBMGA000298‐PA	365	39.4	4.6	1	nscaf1681	1865173	1877267	−
	*CPAP1‐K*	BGIBMGA010077‐PA	782	86.5	8.9	1	nscaf2883	1715789	1723696	+
	*CPAP1‐L*	BGIBMGA001010‐PA	241	27.3	8.7	1	nscaf1898	5651957	5653262	−
	*CPAP1‐M*	BGIBMGA003773‐PA	345	40.8	4.8	1	nscaf2681	98107	113167	−
	*CPAP1‐N*	BGIBMGA010281‐PA	443	45.6	7.5	1	nscaf2986	5452600	5455155	+
	*CPAP1‐O*		*110*	*12.6*	*6.4*	*1*	*nscaf2964*	*689965*	*694164*	+
	*CPAP3‐A1*	BGIBMGA007899‐PA	237	26.4	4.9	3	nscaf2888	5640904	5651579	+
	*CPAP3‐A2*	BGIBMGA007900‐PA	227	25.7	4.6	3	nscaf2888	5667364	5674622	+
	*CPAP3‐B*	BGIBMGA007678‐PA	290	32.6	5.2	3	nscaf2888	5705610	5717891	−
	*CPAP3‐C*	BGIBMGA007677‐PA	262	29.0	4.8	3	nscaf2888	5753062	5767512	−
	*CPAP3‐D1*	BGIBMGA007901‐PA	214	23.7	4.8	3	nscaf2888	5698115	5703866	+
	*CPAP3‐D2*	BGIBMGA007920‐PA	206	23.5	4.9	3	nscaf2888	6686456	669559	+
	*CPAP3‐E1*		712	77.8	5.8	3	nscaf2888	3526446	3522018	−
	*CPAP3‐E2*		280	31.7	4.8	3	nscaf2888	3548427	3535047	−
	*CPAP3‐E3*		237	26.1	5.3	3	nscaf2888	3530902	3529226	+
**Peritrophic matrix proteins (PMPs)**	*PMP1‐A*	BGIBMGA001857‐PA	1276	143.0	8.7	1	nscaf2204	3111886	3116023	−
	*PMP1‐B*	BGIBMGA011851‐PA	539	63.8	3.8	1	nscaf3031	3808300	3812660	+
	*PMP1‐C*	BGIBMGA014488‐PA	412	46.2	8.6	1	nscaffold720	19116	23088	−
	*PMP1‐D*		88	9.9	5.0	1	nscaffold720	46	309	+
	*PMP2‐A*	BGIBMGA009641‐PA	208	23.4	4.9	2	nscaf2964	4356307	4361865	−
	*PMP2‐B*	BGIBMGA001480‐PA	289	31.8	4.6	2	nscaf2136	203020	204799	−
	*PMP2‐C*	BGIBMGA001361‐PA	351	39.3	8.6	2	nscaf2053	26557	30016	−
	*PMP2‐D*	BGIBMGA001491‐PA	222	24.8	5.2	2	nscaf2136	175389	177607	+
	*PMP2‐E*	BGIBMGA007902‐PA	395	44.0	5.4	2	nscaf2888	5740562	5749970	+
	*PMP2‐F*		1044	116.0	5.5	2	nscaf1898	5648095	5651226	+
	*PMP3*	BGIBMGA001504‐PA	342	36.9	4.3	3	nscaf2136	1118346	1120212	+
	*PMP4*		372	37.5	4.4	4	scaffold3918	6	1121	+
	*PMP6*	BGIBMGA000185‐PA	629	64.3	5.0	6	nscaf1289	20223	36914	+
	*PMP12*	BGIBMGA007250‐PA	1606	177.5	4.6	12	nscaf2874	564944	571710	+
	*PMP24*	BGIBMGA009809‐PA	4380	455.2	3.7	24	nscaf2964	4685901	4723941	+
**Chitinases**	*CHT1‐A*	BGIBMGA010240‐PA	543	60.9	5.1	1	nscaf2986	2994945	3009627	+
	*CHT1‐B*		987	111.5	6.2	1	nscaf2829	3360753	3311587	−
	*CHT3*	BGIBMGA006989‐PA	3138	233.5	5.9	3	nscaf2865	4431725	4451466	−
	*CHT7*	BGIBMGA006874‐PA	2723	303.4	5.7	7	nscaf2859	1368402	1392193	+
**Chitin deacetylases**	*CDA1‐A*	BGIBMGA010573‐PA	437	49.7	5.1	1	nscaf2993	6820022	6842885	+
	*CDA1‐B*	BGIBMGA006213‐PA	539	61.4	5.1	1	nscaf2847	8094528	8105043	+
	*CDA2*	BGIBMGA006214‐PA	576	65.3	5.2	2	nscaf2847	8113114	8118608	+

**Figure 1:**
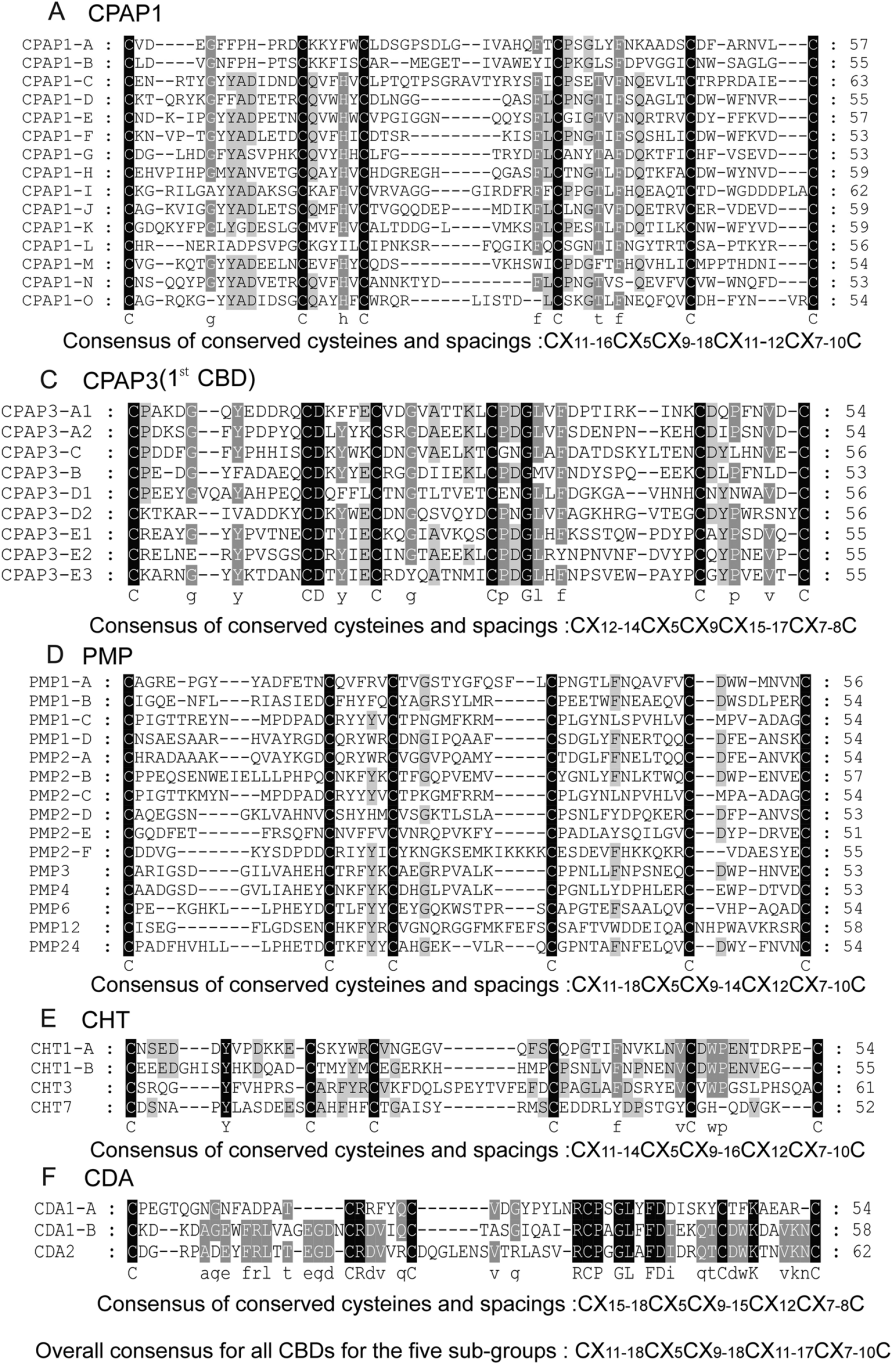
Amino acid sequence alignment of the ChtBD2 of each of the CPAP1, CPAP3, PMP, CHT, and CDA families of *Bombyx mori* proteins. Multiple sequence alignment of ChtBD2s for each of these ChtBD2‐containing proteins was performed using ClustalX software and GeneDoc software. The cysteines and residues conserved in all proteins of that family are shaded in black. (A–F) ClustalX alignment of the ChtBD2 domain from each family of *B. mori* proteins.

Alignment of the CBDs from the proteins in each family showed that the protein sequences of the five categories are highly variable, while six cysteine residues form a highly conserved motif between the five categories. The lengths of the CBDs range from 50 to 63 amino acid residues, when counting from the first to the sixth cysteines. The spacing between cysteines was found to be highly similar; however, the spacing between the other cysteines was greater than that between the second and third cysteines (Fig. 1). The cysteines of the ChtBD2 domain, for each category, are conserved within those of previous reports for other species, including *M. sexta*, *T. castaneum*, and *D. melanogaster* (Behr & Hoch, 2005; Jasrapuria *et al*., 2010; Tetreau *et al*., 2015).

### Gene expression patterns

**Figure 2:**
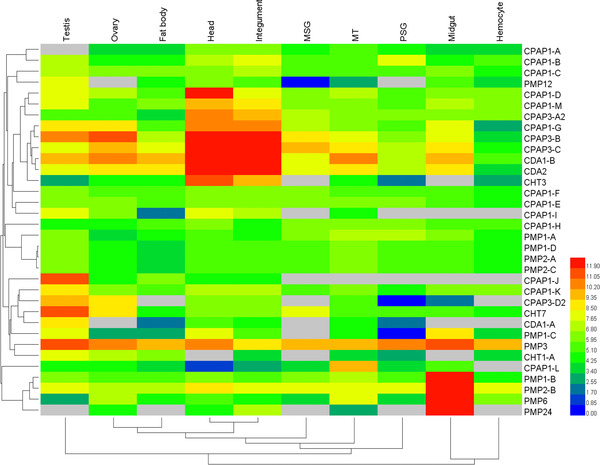
Tissue expression profile of 35 silkworm genes encoding proteins with the ChtBD2 domain. Gene expression levels are represented by red (higher expression) and blue (lower expression) boxes. The columns represent 10 different tissue or organ samples: testis, ovary, head, integument, fat body, midgut, hemocyte, MT (Malpighian tubule), MSG (anterior/median silk gland), and PSG (posterior silk gland).

Microarray analysis of the expression patterns of 35 genes encoding ChtBD2 proteins in *B. mori* showed that most CPAPs were clustered together and expressed in the integument and head. In addition, some CPAP genes were also found to be expressed in the malpighian tubules, MSG (anterior/median silk gland), and PSG (posterior silk gland). Some of the PMPs were clustered together and highly expressed in the midgut, while others exhibited divergent expression patterns with low expression in the hemocyte, testis, ovary and malpighian tubules (Fig. 2).

### Expression profiles and phylogenetic analysis of BmCBP1

**Figure 3:**
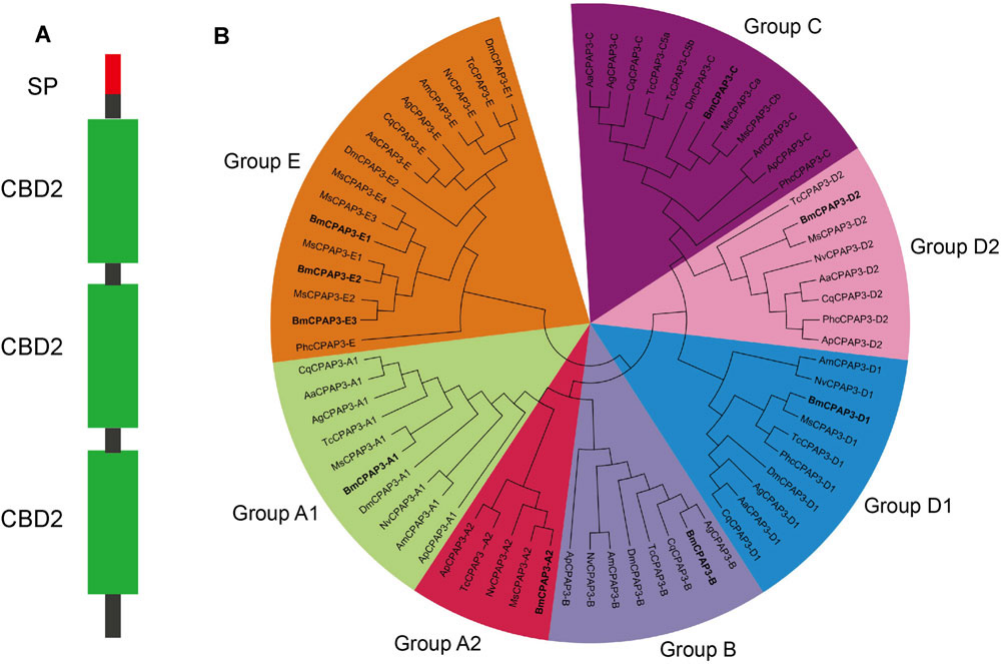
BmCBP1 is a conserved chitin‐binding domain protein within CPAP3 family from different insect species. (A) Diagram showing the signal peptide (SP, red), chitin‐binding domain (CBD2, green), and a C‐terminal stretch of BmCBP1. (B) Phylogenetic analysis of CPAP3 families from different insect species; *Aedes aegypti* (Aa), *Anopheles gambiae* (Ag), *Apis mellifera* (Am), *Acyrthosiphon pisum* (Ap), *Bombyx mori* (Bm), *Culex quinquefasciatus* (Cq), *Drosophila melanogaster* (Dm), *Manduca sexta* (Ms), *Nasonia vitripennis* (Np), *P. h. corporis* (Phc), *Tribolium castaneum* (Tc). The CPAP3s from *B. mori* are indicated in bold. The accession numbers of all the proteins used are listed in Supplementary Table S1. CPAP3s are grouped into seven different groups: group A1 (light green), groups A2 (red), group B (dark gray), group C (dark purple), group D1 (dark blue), group D2 (pink) and group E (orange).

In order to further examine the function of cuticle‐related genes with the ChtBD2 domain in silkworm, BmCBP1 (BmCPAP3‐A2), one of the CPAP3s, which showed high expression in the epidermis and accumulated in molt fluid during metamorphosis (Qu *et al*., 2014), was characterized in this study and its expression and localization was investigated using molecular tools. The result showed that BmCBP1 possesses a signal peptide and three ChtBD2 domains, spaced by two linker regions (Fig. 3A). Phylogenetic analysis showed the CPAP3 family has conserved sequences within different species, including *M. sexta*, *Nasonia vitripennis*, *T. casteneum*, *Apis mellifera*, *D. melanogaster* as well as *P. h. corporis*. A total of nine BmCPAP3 proteins from the *B. mori* genome were divided into seven phylogenetic groups of CPAP3 family from different species. BmCBP1 (BmCPAP3‐A2) was observed in Group A2 of the phylogenetic tree (Fig. 3B).

**Figure 4:**
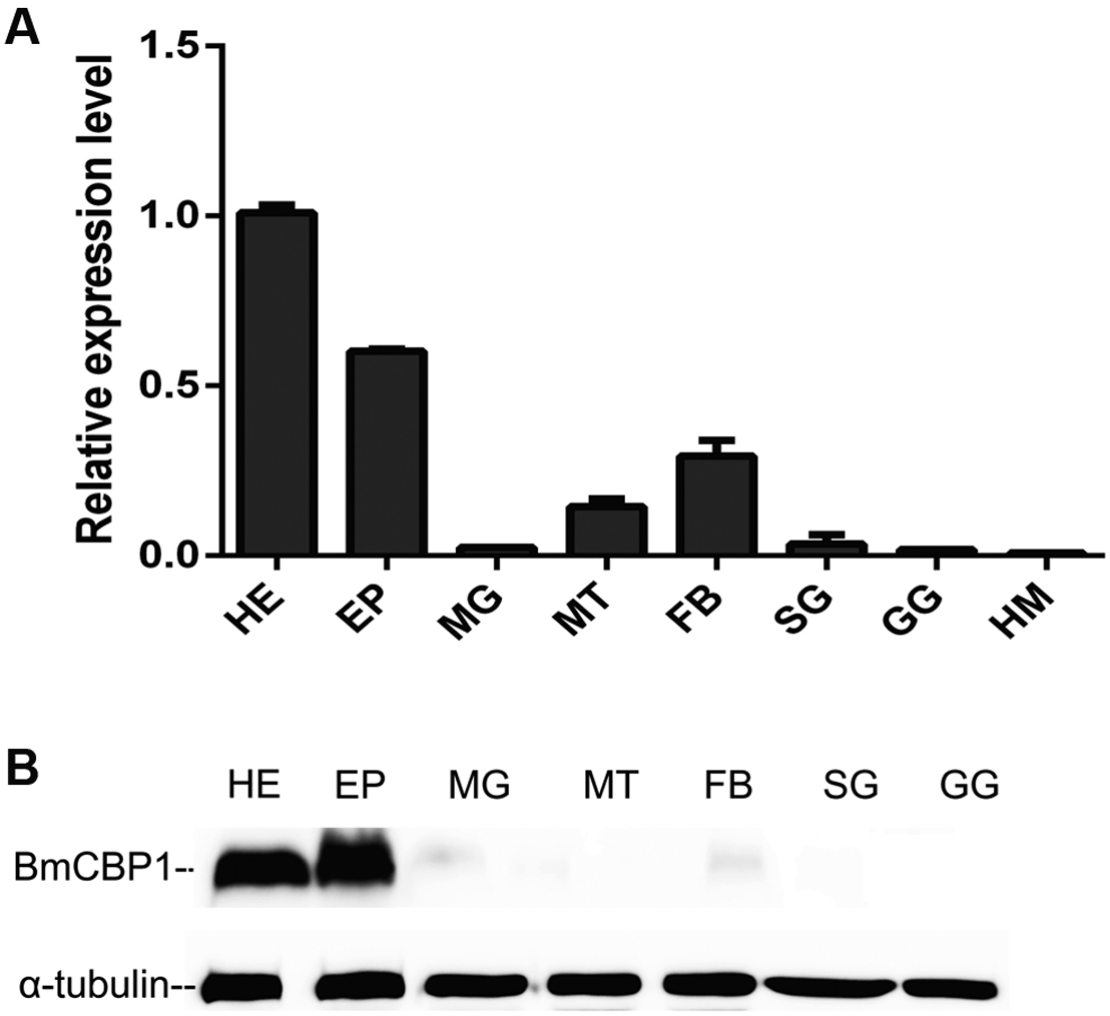
Analysis of BmCBP1 in various prepupal tissues. (A) Quantitative polymerase chain reaction analysis of *BmCBP1* mRNA in various prepupal tissues. (B) Western blot analysis of BmCBP1 protein in various prepupal tissues: HE, head; EP, epidermis; MG, midgut; MT, Malpighian tubule; FB, fat body; SG, silk gland; GG, genital gland; HM, hemolymph.

The ChtBD2 chitin‐binding domain may act as a conserved basic module for BmCBP1, and its homologs may play a role in the organization of the chitinous cuticle (Petkau *et al*., 2012). The data from quantitative PCR showed that *BmCBP1* is mainly expressed in the larval head and epidermis, and weakly expressed in the fat bodies (Fig. 4A). The distribution of protein among the tissues was consistent with the mRNA expressional profiles, as indicated by western blot analysis (Fig. 4B).

### Expression patterns of BmCBP1 in the epidermis at various developmental stages

**Figure 5:**
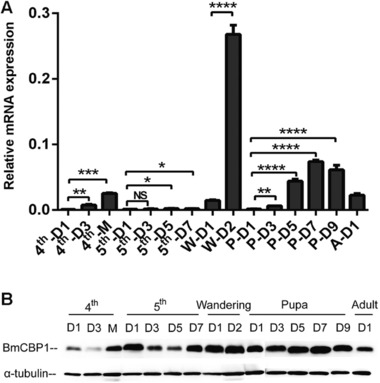
Expression profile of BmCBP1 in the epidermis at various developmental stages. (A) Quantitative polymerase chain reaction analysis of *BmCBP1* messenger RNA in epidermis from 4th instar larval stage to 1st day of moth: Dn, day n; 4th, fourth instar larval stage; 5th, fifth instar larval stage; W, wandering stage; P, pupa; A, adult. (B) Analysis of BmCBP1 protein in the epidermis, from 4th instar larval stage to 1st day of moth by western blot: Dn, day n; 4th, fourth instar larval stage; 5th, fifth instar larval stage.

RNA and protein extracts were obtained from the epidermis to perform a temporal expression analysis of the 4th instar larvae to the moth stages. The results demonstrated *BmCBP1* exhibits varied expression during the developmental stages, with dramatic upregulation of expression just before ecdysis and metamorphosis, during molting day of 4th instar, 2nd day of wandering stage, and the late pupal stage. Furthermore, the level of protein expression was consistent with that of mRNA expression. Similar patterns were confirmed at the developmental stages, from 1st day of 4th instar to day of eclosion, via western blotting (Fig. 5).

### The responses of the BmCBP1 gene to 20E

**Figure 6:**
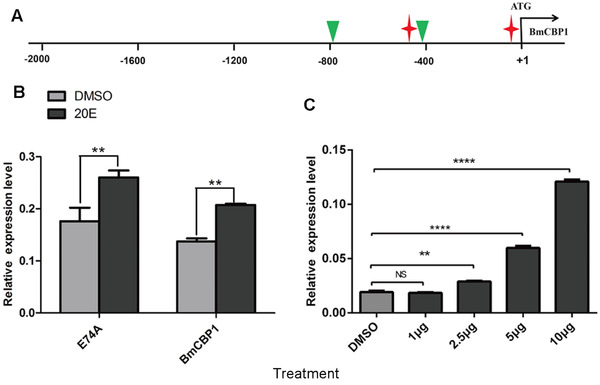
The responses of the *BmCBP1* gene to 20‐hydroxyecdysone (20E). (A) Putative ecdysone response elements of EcR::usp and Eip74EF in the promoter of *BmCBP1* (red asterisk, EcR::usp; green triangle, Eip74EF). (B) Quantitative polymerase chain reaction analysis of the expression of the *BmCBP1* gene in silkworms induced by 2.5 μg of 20E. E74A, ecdysone inducible transcription factor. (C) Quantitative polymerase chain reaction analysis of the expression of the *BmCBP1* gene in the silkworm induced by different concentrations of 20E. DMSO; dimethyl sulfoxide.

We performed bioinformatics analysis on the upstream promoter sequences of *BmCBP1* genes. Some putative ecdysone response elements including two elements of EcR::usp (−542 to −528; −35 to −21) and two elements of Eip74EF (−769 to −763; −506 to −500) were identified from the promoter sequence of BmCBP1 (Fig. 6A). In order to identify whether 20E could affect the expression of *BmCBP1* gene *in vivo*, 20E treatment was conducted. Quantitative reverse transcription PCR results for larval epidermis showed that *BmCBP1* was notably up‐regulated after 24 h by 20E treatment compared to the control, and expression of *BmCBP1* gene increased gradually with the increase of the 20E injection, which suggested that *BmCBP1* was regulated by 20E during molting (Fig. 6B–C).

### Immunofluorescence co‐localization of chitin and BmCBP1 in the cuticle

**Figure 7:**
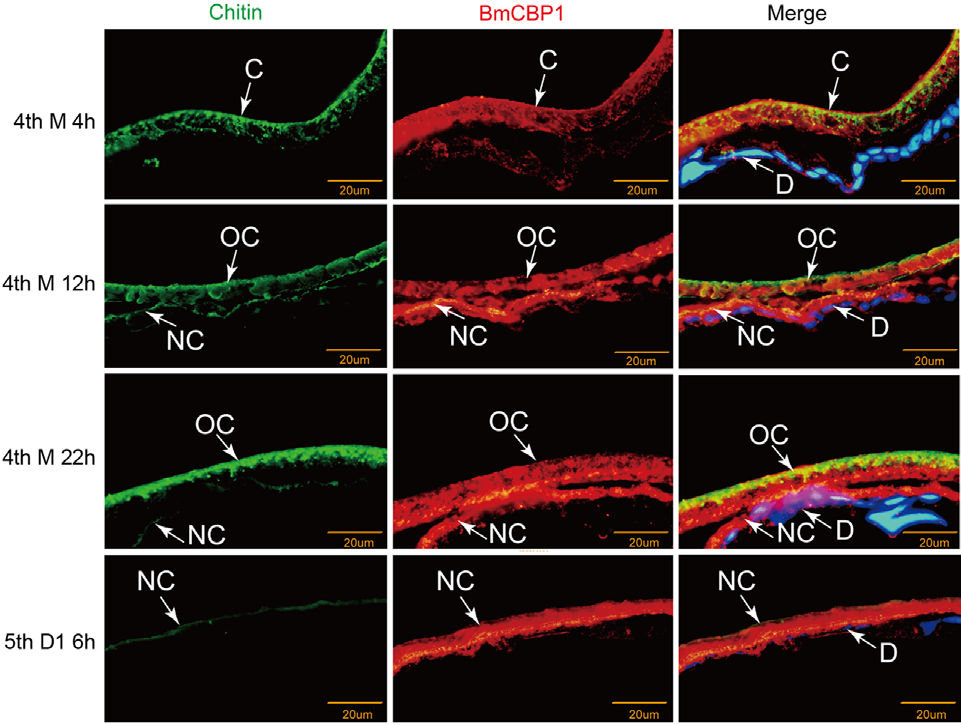
Immunofluorescence analysis of co‐localization of chitin and BmCBP1 in the cuticle during the 4th molt. Cross‐sections (5 μm) were treated with anti‐BmCBP1 (red). Fluorescein isothiocyanate‐conjugated wheat germ agglutinin (WGA) was used to stain cuticle chitin (green). Nuclei were stained with 4′,6‐diamidino‐2‐phenylindole (blue) and the sections were observed by fluorescence microscopy. 4th M 4 h: 4 hours of 4th molt; 4th M 12 h: 12 hours of 4th molt; 4th M 22 h: 22 hours of 4th molt; 5th D1 6 h: 6 hours of 1st day in 5th instar: C: cuticle; OC: old cuticle; NC: new cuticle; D: dermis.

In order to explore the function of BmCBP1 during the development of the silkworm, we additionally performed co‐localization of BmCBP1 protein and chitin from the 4th instar larval molting stage to the 1st day of 5th instar. At the early stage of the 4th molt of silkworm larva, the chitin showed a clear distribution in the cuticle. As development progressed, the new cuticle acquired shape and a weak chitin signal appeared under the old cuticle. The BmCBP1 protein demonstrated strong expression in the old cuticle and a faint signal during the early stage of molting. The expression of this protein was upregulated during the formation of the new cuticle, at 12 h of the 4th molt. Prior to ecdysis in the silkworm, the chitin was highly enriched in the outer sphere of the old cuticle. Strong expression of BmCBP1 could be detected at 22 h of the 4th molt. Immunofluorescence data revealed that chitin and BmCBP1 shared similar expression patterns in the epidermis throughout molting of the 4th instar. The uniform distribution of BmCBP1 and chitin in the entire cuticle, and their relative abundance, suggested that BmCBP1 participated in the formation of the new cuticle during molting (Fig. 7).

## Discussion

In this study, we performed a comprehensive search of the silkworm genome for genes encoding proteins that contain ChtBD2 chitin‐binding domains. Our results indicated that six cysteines of the ChtBD2 chitin‐binding domain, for each category, are conserved in insects such as *M. sexta* and *T. castaneum* (Jasrapuria *et al*., 2010; Tetreau *et al*., 2015). Compared with the 46 chitin‐binding proteins in silkworm, 56 chitin‐binding proteins are found in *M. sexta*, which include 26 CPAPs, 17 PMPs, six chitinases and seven chitin deacetylases. Fifty chitin‐binding proteins have been identified in *T. castaneum*, which comprise 18 CPAPs, 11 PMPs, 13 chitinases and chitin deacetylases, and the remaining eight proteins are classified as miscellaneous proteins (Jasrapuria *et al*., 2010; Tetreau *et al*., 2015). The CPAP1 family and CPAP3 family also have been found in *M. sexta* and *T. cast*aneum and the CPAP family may be derived from a common ancestor (Jasrapuria *et al*., 2010; Tetreau *et al*., 2015). The ChtBD2 chitin‐binding domain, which is considered to act as a basic module that is acquired by other proteins, including enzymes, is involved in chitin metabolism to modify their function (Tetreau *et al*., 2015). It is possible that a higher number of ChtBD2 leads to binding to the chitin (Jasrapuria *et al*., 2010).

The homologs of the CPAP‐encoding genes were expressed in the head and epidermis in the tissues of the 3rd day of the 5th instar (Fig. 2). The spatial expression profiles additionally showed that BmCBP1 is mainly expressed in the head and epidermis (Fig. 4). However, the expression pattern of the PMP gene family differed from that of the CPAPs, which are mainly expressed in the midgut, although they have high similarity to and share conserved sequence and domains with the CPAP family. Elvin *et al*. reported that multiple cysteine‐rich domains in peritrophin‐44 are responsible for binding to chitin. It was additionally reported that the major structural protein of the peritrophic membrane may play a role in the maintenance of the peritrophic membrane structure and porosity (Elvin *et al*., 1996). Although the CPAP and PMP families possess similar domains, they differ in terms of function.

Several cuticle‐related proteins were identified from molting fluid in 2014, including chitinase, hexosaminides, and several CPAP proteins (Qu *et al*., 2014). BmCBP1, one of these cuticle‐related proteins, exhibited mostly an eight‐fold increase in expression at the pupal–adult stage compared to at the larval–pupal stage (Qu *et al*., 2014). In this study, we found that expression of *BmCBP1* increased obviously before larva–pupa and pupa–adult metamorphosis. However, the highest expression of *BmCBP1* occurred in the larval–pupa rather than pupal–adult stages of metamorphism in the quantitative PCR analyses, which was different from the proteomic result using the molting fluid (Qu *et al*., 2014). Constant accumulation of BmCBP1 from the 1st day to 9th day of the pupal stage was observed on western blotting. Therefore, we presumed that BmCBP1 protein was expressed in the cuticle during the whole pupal stage and secreted in the molting fluid of the silkworm before eclosion.

*BmCBP1* was upregulated during the wandering stage and late‐pupal stage, which is constant with the change of ecdysone titer in silkworm (Mizoguchi *et al*., 2002). The results from the 20E treatment also confirmed that *BmCBP1* was dramatically upregulated by 20E. It has been reported that the promoter region of the cuticle protein possesses ecdysone response elements that mediate the response to ecdysone under regulation by ecdysone‐responsive transcription factors (Wang *et al*., 2009; Ali *et al*., 2012; Akagi *et al*., 2013). Bioinformatics analysis of the upstream promoter sequences of *BmCBP1* genes revealed two putative EcR::usp and two Eip74EF elements in the *BmCBP1* promoter. In future studies, the putative elements will be deleted or mutated to determine which elements are responsible for the response to 20E in the *BmCBP1* promoter.

It has been reported that proteins containing the ChtBD2 domain have chitin‐binding activity (Elvin *et al*., 1996; Tang *et al*., 2010; Petkau *et al*., 2012; Chen *et al*., 2014; Dong *et al*., 2016; Tajiri *et al*., 2017). In this study, we demonstrated that BmCBP1 and chitin co‐localized in the epidermal layer, and that the emergence of the BmCBP1 signal in the new cuticle appeared slightly earlier than the chitin signal in the new cuticle. These results indicate that BmCBP1 is an important structural component of the new cuticle. In *Drosophila*, obstructor‐A, a member of CPAP3 family, was confirmed to be involved in the extracellular matrix in cuticle‐forming organs. Loss of obstructor‐A led to severe defects during cuticle molting and tube expansion (Petkau *et al*., 2012; Pesch *et al*., 2015). In fact, we also performed RNA interference (RNAi) for the gene encoding *BmCBP1* in the silkworm at the 4th instar of larvae. However, obvious differences of phenotype were not observed between RNAi for *BmCBP1* and the control (Fig. S1). Many RNAi studies have been tried in Lepidoptera; however, some of them proved difficult to achieve (Terenius *et al*., 2011). The high expression of *BmCBP1* during metamorphosis may lead to less obvious differences of phenotype in RNAi experiments. We think the gene knockout may be a more effective tool for studying the function of the *BmCBP1* gene in silkworm.

## Disclosure

The authors declare no conflict of interest.

## Supporting information


**Fig S1**. Analysis of RNAi mediated by dsRNAs for *BmCBP1* genes. Phenotypes produced by injection of dsRNAs for *BmCBP1* genes and control (GFP) B. Q‐PCR analyses of target specificity of RNAi mediated by dsRNAs for *BmCBP1* genes. C. Western blot analyses of target specificity of RNAi mediated by dsRNAs for *BmCBP1* genes.

**Table S1**. Accession numbers of CPAP3 family from different species.

## References

[ins12552-bib-0001] Akagi, K. , Kageyama, Y. , Kayashima, Y. , Takakura, Y. , Hirose, S. and Ueda, H. (2013) The binding of multiple nuclear receptors to a single regulatory region is important for the proper expression of EDG84A in *Drosophila melanogaster* . Journal of Molecular Biology, 425, 71–81. 23137796 10.1016/j.jmb.2012.10.020

[ins12552-bib-0002] Ali, M.S. , Iwanaga, M. and Kawasaki, H. (2012) Ecdysone‐responsive transcription factors determine the expression region of target cuticular protein genes in the epidermis of *Bombyx mori* . Development Genes and Evolution, 222, 89–97. 22460818 10.1007/s00427-012-0392-x

[ins12552-bib-0003] Andersen, S.O. , Hojrup, P. and Roepstorff, P. (1995) Insect cuticular proteins. Insect Biochemistry and Molecular Biology, 25, 153–176. 7711748 10.1016/0965-1748(94)00052-j

[ins12552-bib-0004] Behr, M. and Hoch, M. (2005) Identification of the novel evolutionary conserved obstructor multigene family in invertebrates. FEBS Letters, 579, 6827–6833. 16325182 10.1016/j.febslet.2005.11.021

[ins12552-bib-0005] Bradford, M.M. (1976) A rapid and sensitive method for the quantitation of microgram quantities of protein utilizing the principle of protein‐dye binding. Analytical Biochemistry, 72, 248–254. 942051 10.1016/0003-2697(76)90527-3

[ins12552-bib-0006] Chang, T.C. and Stergiopoulos, I. (2015) Inter‐ and intra‐domain horizontal gene transfer, gain‐loss asymmetry and positive selection mark the evolutionary history of the CBM14 family. FEBS Journal, 282, 2014–2028. 25754577 10.1111/febs.13256

[ins12552-bib-0007] Chen, W.J. , Huang, L.X. , Hu, D. , Liu, L.Y. , Gu, J. , Huang, L.H. *et al* (2014) Cloning, expression and chitin‐binding activity of two peritrophin‐like protein genes in the common cutworm, Spodoptera litura. Insect Science, 21, 449–458. 23955994 10.1111/1744-7917.12055

[ins12552-bib-0008] Deng, H.M. , Li, Y. , Zhang, J.L. , Liu, L. and Feng, Q.L. (2016) Analysis of expression and chitin‐binding activity of the wing disc cuticle protein BmWCP4 in the silkworm, Bombyx mori. Insect Science, 23, 782–790. 25953667 10.1111/1744-7917.12231

[ins12552-bib-0009] Deng, W.K. , Wang, Y.B. , Liu, Z.X. , Cheng, H. and Xue, Y. (2014) HemI: a toolkit for illustrating heatmaps. PLoS ONE, 9, e111988. 25372567 10.1371/journal.pone.0111988 PMC4221433

[ins12552-bib-0010] Dong, Z.M. , Zhang, W.W. , Zhang, Y. , Zhang, X.L. , Zhao, P. and Xia, Q.Y. (2016) Identification and characterization of novel chitin‐binding proteins from the larval cuticle of silkworm, Bombyx mori. Journal Proteome Research, 15, 1435–1445. 10.1021/acs.jproteome.5b00943 26972338

[ins12552-bib-0011] Elvin, C.M. , Vuocolo, T. , Pearson, R.D. , East, I.J. , Riding, G.A. , Eisemann, C.H. *et al* (1996) Characterization of a major peritrophic membrane protein, peritrophin‐44, from the larvae of *Lucilia cuprina* cDNA and deduced amino acid sequences. Journal of Biological Chemistry, 271, 8925–8935. 8621536 10.1074/jbc.271.15.8925

[ins12552-bib-0012] Futahashi, R. , Okamoto, S. , Kawasaki, H. , Zhong, Y.S. , Iwanaga, M. , Mita, K. *et al* (2008) Genome‐wide identification of cuticular protein genes in the silkworm, *Bombyx mori* . Insect Biochemistry and Molecular Biology, 38, 1138–1146. 19280704 10.1016/j.ibmb.2008.05.007

[ins12552-bib-0013] Hamodrakas, S.J. , Willis, J.H. and Iconomidou, V.A. (2002) A structural model of the chitin‐binding domain of cuticle proteins. Insect Biochemistry and Molecular Biology, 32, 1577–1583. 12530225 10.1016/s0965-1748(02)00079-6

[ins12552-bib-0014] Hegedus, D. , Erlandson, M. , Gillott, C. and Toprak, U. (2009) New insights into peritrophic matrix synthesis, architecture, and function. Annual Reviews of Entomology, 54, 285–302. 10.1146/annurev.ento.54.110807.090559 19067633

[ins12552-bib-0015] Iconomidou, V.A. , Willis, J.H. and Hamodrakas, S.J. (2005) Unique features of the structural model of ‘hard’ cuticle proteins: implications for chitin‐protein interactions and cross‐linking in cuticle. Insect Biochemistry and Molecular Biology, 35, 553–560. 15857761 10.1016/j.ibmb.2005.01.017

[ins12552-bib-0016] Jasrapuria, S. , Arakane, Y. , Osman, G. , Kramer, K.J. , Beeman, R.W. and Muthukrishnan, S. (2010) Genes encoding proteins with peritrophin A‐type chitin‐binding domains in *Tribolium castaneum* are grouped into three distinct families based on phylogeny, expression and function. Insect Biochemistry and Molecular Biology, 40, 214–227. 20144715 10.1016/j.ibmb.2010.01.011

[ins12552-bib-0017] Jasrapuria, S. , Specht, C.A. , Kramer, K.J. , Beeman, R.W. and Muthukrishnan, S. (2012) Gene families of cuticular proteins analogous to peritrophins (CPAPs) in *Tribolium castaneum* have diverse functions. PLoS ONE, 7, e49844. 23185457 10.1371/journal.pone.0049844 PMC3504105

[ins12552-bib-0018] Mizoguchi, A. , Dedos, S.G. , Fugo, H. and Kataoka, H. (2002) Basic pattern of fluctuation in hemolymph PTTH titers during larval–pupal and pupal–adult development of the silkworm, Bombyx mori. Genernal and Comparative Endocrinology, 127, 181–189. 10.1016/s0016-6480(02)00043-6 12383446

[ins12552-bib-0019] Mun, S. , Young Noh, M. , Dittmer, N.T. , Muthukrishnan, S. , Kramer, K.J. , Kanost, M.R. *et al* (2015) Cuticular protein with a low complexity sequence becomes cross‐linked during insect cuticle sclerotization and is required for the adult molt. Scientific Reports, 5, 10484. 25994234 10.1038/srep10484 PMC4440208

[ins12552-bib-0020] Pesch, Y.Y. , Riedel, D. and Behr, M. (2015) Obstructor A organizes matrix assembly at the apical cell surface to promote enzymatic cuticle maturation in *Drosophila* . Journal of Biological Chemistry, 290, 10071–10082. 25737451 10.1074/jbc.M114.614933 PMC4400323

[ins12552-bib-0021] Petkau, G. , Wingen, C. , Jussen, L.C. , Radtke, T. and Behr, M. (2012) Obstructor‐A is required for epithelial extracellular matrix dynamics, exoskeleton function, and tubulogenesis. Journal of Biological Chemistry, 287, 21396–21405. 22544743 10.1074/jbc.M112.359984 PMC3375561

[ins12552-bib-0022] Qu, M. , Ma, L. , Chen, P. and Yang, Q. (2014) Proteomic analysis of insect molting fluid with a focus on enzymes involved in chitin degradation. Journal of Proteome Research, 13, 2931–2940. 24779478 10.1021/pr5000957

[ins12552-bib-0023] Rebers, J.E. and Riddiford, L.M. (1988) Structure and expression of a *Manduca sexta* larval cuticle gene homologous to *Drosophila* cuticle genes. Journal of Molecular Biology, 203, 411–423. 2462055 10.1016/0022-2836(88)90009-5

[ins12552-bib-0024] Rebers, J.E. and Willis, J.H. (2001) A conserved domain in arthropod cuticular proteins binds chitin. Insect Biochemistry and Molecular Biology, 31, 1083–1093. 11520687 10.1016/s0965-1748(01)00056-x

[ins12552-bib-0025] Schorderet, S. , Pearson, R.D. , Vuocolo, T. , Eisemann, C. , Riding, G.A. and Tellam, R.L. (1998) cDNA and deduced amino acid sequences of a peritrophic membrane glycoprotein, ‘peritrophin‐48’, from the larvae of *Lucilia cuprina* . Insect Biochemistry and Molecular Biology, 28, 99–111. 9639876 10.1016/s0965-1748(97)00103-3

[ins12552-bib-0026] Shen, Z. and Jacobs‐Lorena, M. (1998) A type I peritrophic matrix protein from the malaria vector *Anopheles gambiae* binds to chitin. Cloning, expression, and characterization. Journal of Biological Chemistry, 273, 17665–17670. 9651363 10.1074/jbc.273.28.17665

[ins12552-bib-0027] Tajiri, R. , Ogawa, N. , Fujiwara, H. and Kojima, T. (2017) Mechanical control of whole body shape by a single cuticular protein obstructor‐E in *Drosophila melanogaster* . PLoS Genet, 13. 10.1371/journal.pgen.1006548 PMC5226733 28076349

[ins12552-bib-0028] Tang, L. , Liang, J.B. , Zhan, Z.G. , Xiang, Z.H. and He, N.J. (2010) Identification of the chitin‐binding proteins from the larval proteins of silkworm, *Bombyx mori* . Insect Biochemistry and Molecular Biology, 40, 228–234. 20149871 10.1016/j.ibmb.2010.01.010

[ins12552-bib-0029] Tellam, R.L. , Wijffels, G. and Willadsen, P. (1999) Peritrophic matrix proteins. Insect Biochemistry and Molecular Biology, 29, 87–101. 10196732 10.1016/s0965-1748(98)00123-4

[ins12552-bib-0030] Terenius, O. , Papanicolaou, A. , Garbutt, J.S. , Eleftherianos, I. , Huvenne, H. , Kanginakudru, S. *et al* (2011) RNA interference in Lepidoptera: an overview of successful and unsuccessful studies and implications for experimental design. Journal of Insect Physiology, 57, 231–245. 21078327 10.1016/j.jinsphys.2010.11.006

[ins12552-bib-0031] Tetreau, G. , Dittmer, N.T. , Cao, X. , Agrawal, S. , Chen, Y.R. , Muthukrishnan, S. *et al* (2015) Analysis of chitin‐binding proteins from *Manduca sexta* provides new insights into evolution of peritrophin A‐type chitin‐binding domains in insects. Insect Biochemistry and Molecular Biology, 62, 127–141. 25524298 10.1016/j.ibmb.2014.12.002 PMC9346963

[ins12552-bib-0032] Togawa, T. , Nakato, H. and Izumi, S. (2004) Analysis of the chitin recognition mechanism of cuticle proteins from the soft cuticle of the silkworm, Bombyx mori. Insect Biochemistry and Molecular Biology, 34, 1059–1067. 15475300 10.1016/j.ibmb.2004.06.008

[ins12552-bib-0033] Wang, H.B. , Nita, M. , Iwanaga, M. and Kawasaki, H. (2009) betaFTZ‐F1 and broad‐complex positively regulate the transcription of the wing cuticle protein gene, BMWCP5, in wing discs of *Bombyx mori* . Insect Biochemistry and Molecular Biology, 39, 624–633. 19580866 10.1016/j.ibmb.2009.06.007

[ins12552-bib-0034] Wang, P. and Granados, R.R. (2001) Molecular structure of the peritrophic membrane (PM): identification of potential PM target sites for insect control. Archives of Insect Biochemistry Physiology, 47, 110–118. 11376457 10.1002/arch.1041

[ins12552-bib-0035] Xia, Q.Y. , Cheng, D.J. , Duan, J. , Wang, G.H. , Cheng, T.C. , Zha, X.F. *et al* (2007) Microarray‐based gene expression profiles in multiple tissues of the domesticated silkworm, Bombyx mori. Genome Biology, 8, R162. 17683582 10.1186/gb-2007-8-8-r162 PMC2374993

