# Supplementary figures and images for: Genome‐wide identification of chitin‐binding proteins and characterization of BmCBP1 in the silkworm, Bombyx mori
Source: Insect Sci. 2018 Feb 4;26(3):400–12. doi: 10.1111/1744-7917.12552 (PMC7379184; doi:10.1111/1744-7917.12552)

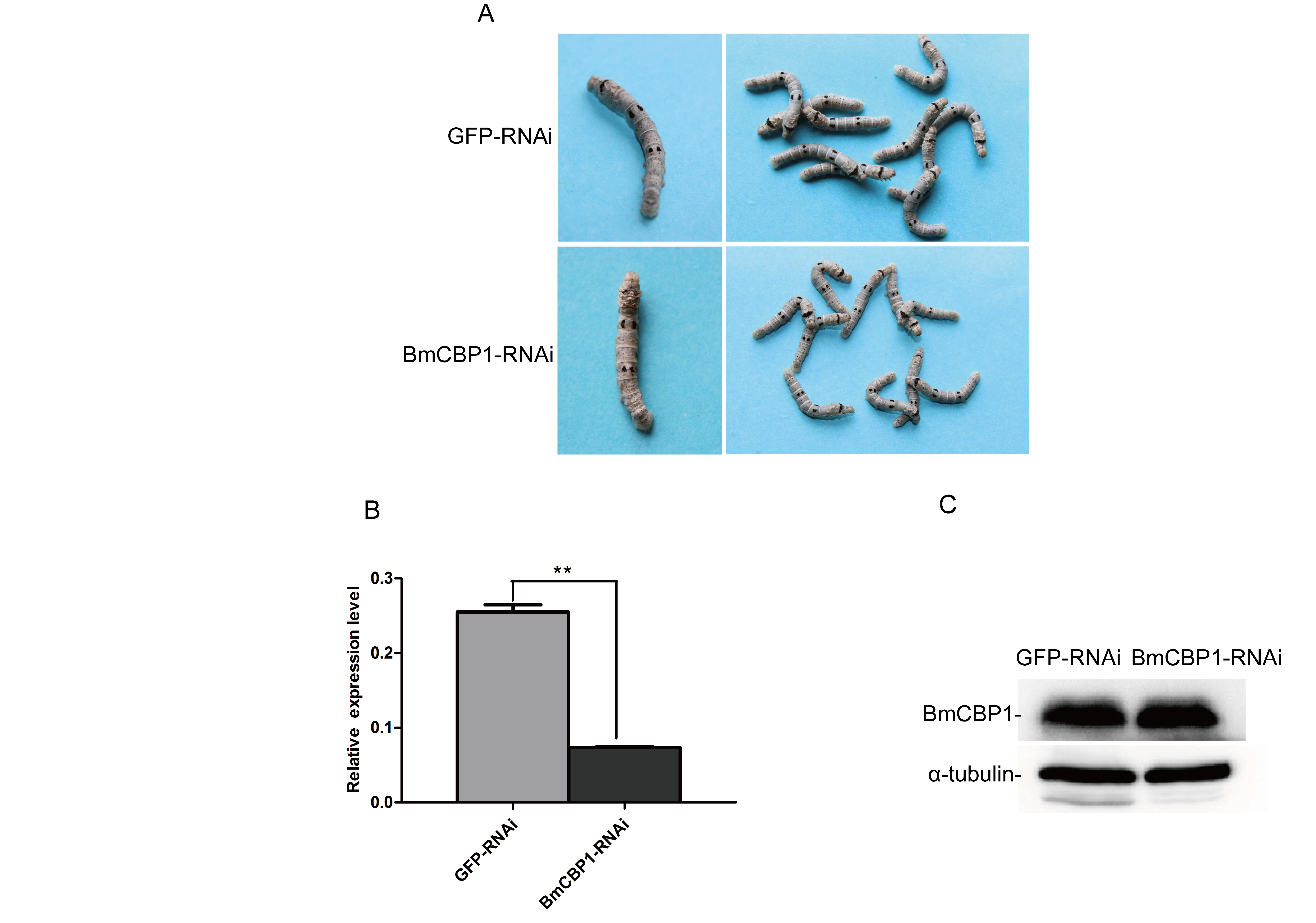

Supplement: Supplementary file 1 — Fig S1. Analysis of RNAi mediated by dsRNAs for BmCBP1 genes. Phenotypes produced by injection of dsRNAs for BmCBP1 genes and control (GFP) B. Q‐PCR analyses of target specificity of RNAi mediated by dsRNAs for BmCBP1 genes. C. Western blot analyses of target specificity of RNAi mediated by dsRNAs for BmCBP1 genes. [file INS-26-400-s001.tif]
